# On the Role of Damage Evolution in Finite Element Modeling of the Cutting Process and Sensing Residual Stresses

**DOI:** 10.3390/s22218547

**Published:** 2022-11-06

**Authors:** Mohamed M. A. Ammar, Bijan Shirinzadeh, Hassan Elgamal, Mohamed N. A. Nasr

**Affiliations:** 1 Department of Mechanical Engineering, Faculty of Engineering, Alexandria University, Alexandria 5424041, Egypt; 2 Robotics and Mechatronics Research Laboratory, Department of Mechanical and Aerospace Engineering, Monash University, Clayton 3800, Australia; 3 Structural Engineering, Compressor Static Structures, Pratt & Whitney, Longueuil, QC J4G 1A1, Canada

**Keywords:** sensing, finite element modeling (FEM), machining, damage modeling, residual stresses

## Abstract

This study focuses on the role of the damage evolution when estimating the failure behavior of AISI 1045 steel for sensing and measuring metal cutting parameters. A total of five Lagrangian explicit models are established to investigate the effect of applying damage evolution techniques. The Johnson–Cook failure model is introduced once to fully represent damage behavior, i.e., no damage evolution is considered, and as a damage initiation criterion in the remaining approaches. A fracture energy-based model is included to model damage propagation with different evolution rates. Temperature-dependent and temperature-independent fracture energy models are also investigated. Dry orthogonal cutting and residual stresses measurements of AISI 1045 are conducted for validation. The significance of the damage evolution is investigated using honed-tool and sharp-tool models. Including the damage evolution led to a prediction of higher workpiece temperatures, plastic strains, cutting forces, and residual stresses, with no clear differences between linear and exponential evolution rates. The role of damage evolution is more evident when temperature-dependent evolution models are used.

## 1. Introduction

The use of finite element modeling (FEM) to simulate metal cutting has been on the rise over the past three decades. FEM has been used in a wide variety of metal cutting applications; these include cryogenic machining [1,2,3], laser-assisted machining (LAM) [4,5], and sequential cutting [6], as examples. The currently available FEM techniques include Eulerian cutting models, which can be found in [7,8]; Lagrangian models such as [9,10,11,12,13,14]; adaptive Lagrangian–Eulerian (ALE) models, which were implemented in [15,16,17]; coupled Eulerian–Lagrangian (CEL) formulation, as implemented in [18,19,20]; and, finally, examples of utilizing smoothed particle hydrodynamics (SPH), which can be found in [4,21,22].

In a Lagrangian cutting model, the chip thickness needs to be predefined and a sacrificial layer is required to separate the predefined chip from the to-be-generated machined surface. To generate the chip, a failure criterion (damage model) needs to be defined within the sacrificial layer. Upon the satisfaction of such a criterion, the sacrificial layer material fails to separate the chip from the machined surface. It is important to note that the sacrificial layer, which is sometimes referred to as a parting line, represents a loss of the material volume that does not exist in reality [23]. Accordingly, the parting line should be kept small as much as possible to avoid any deviation in the numerical results. A damage model/failure criterion is also required to be defined within the chip to simulate segmentation, in case such a phenomenon exists. Different damage models were used in the literature, where the Johnson–Cook (J–C) failure criterion is one of the most widely implemented models on ductile metals [24]. The J–C damage model depends on the magnitude of the equivalent plastic strain (${\bar{\varepsilon }}^{pl}$), where damage occurs when ${\bar{\varepsilon }}^{pl}$ reaches a predefined failure strain value (${\bar{\varepsilon }}_{f}^{pl}$) [25]. Originally, the J–C failure criterion was used to entirely simulate the damage process, which results in sudden failure once the criterion is met [26,27]. However, recently, its usage has been limited to model damage initiation and a different damage model has been used to simulate damage evolution. Damage evolution criteria that have been utilized are based on Hillerborg’s fracture energy criterion [28,29,30,31]. The use of damage evolution criteria was reported to provide a smooth degradation of the material and to improve the computational stability [28,29,30].

Mabrouki et al. [11] used FEM to examine the effects of cutting speed on chip formation during dry orthogonal cutting of A2024-T351 aluminum alloy. In their work, the authors used the J–C failure model to initiate chip generation and a fracture energy-based model to simulate the damage evolution and stiffness degradation. They applied a linear evolution rate within the parting line and an exponential evolution rate within the chip. Based on the various deformation mechanisms involved in chip generation and segmentation, mode I and mode II fracture energies were defined within the parting line and the chip, respectively. The experimental cutting results supported the chip morphology prediction, which demonstrated the implementation of the utilized damage evolution model. Zhang et al. [10] developed a finite element (FE) model to simulate the dry orthogonal cutting of Ti–6Al–4V titanium, with a special focus on the tool–chip contact behavior. For damage modeling, the J–C damage model was used as an initiation criterion and an energy-based criterion was used for damage evolution. Mode I fracture was applied through the parting line, along with an exponential evolution rate, while a mode II fracture with a linear evolution rate was used within the chip. The numerical results were compared to experimental data obtained from the literature and a good agreement was found.

Chen et al. [32] presented an analytical flow stress model to predict the flow stress of Al7075-T6 aluminum alloy during the machining process. The model accounted for material plastic flow including a failure criterion and was implemented in FE simulations. The authors reported an improved version of the J–C damage model that accounts for temperature, pressure, stress triaxiality, and strain rate effects, and used it as a damage initiation criterion. An energy density-based ductile failure criterion was also utilized to predict the flow stress during failure as a representation of failure evolution. An exponential damage evolution rate was assumed, where mode I and mode II fractures were implemented within the parting line and chip, respectively. The authors examined the effect of failure initiation strain on the tool–chip contact and validated their predictions using the cutting forces and tool–chip contact length, which was measured experimentally. They continued their work in [30] by developing a high-speed machining FE model of Ti–6Al–4V titanium alloy. The modified J–C failure model utilizing an energy-based ductile failure criterion was also applied. The linear evolution rate was implemented in the sacrificial layer and chip region. Good agreement was found between the predicted cutting forces, as well as chip morphology and the corresponding experimental results. In their orthogonal cutting FE simulations of Ti–6Al–4V titanium alloy, Wang and Liu [33] implemented linear damage evolution that considered the fracture energy, along with the J–C failure model for modeling damage initiation. Good agreement was found between the simulated and experimental results, where chip serration was found to increase with cutting speed.

Abushawashi et al. [29] developed a set of FE models to simulate the cutting process of steel AISI 1045. Damage evolution, which considered the material fracture energy, with mode I within the parting line and mode II within the chip, was implemented, and an exponential evolution rate was assumed in both regions. A case with no evolution was also modeled. The chip characteristics were predicted and compared to the experimental findings and damage evolution was identified to play an important role in the cutting simulation. The authors advised that the post damage evolution should be integrated into the failure model for smooth material degradation and the enhancement of computational stability.

Machining-induced residual stresses (RSs) are an inherent by-product of any machining process. They result from non-uniform plastic deformation and/or phase transformation and have a profound impact on the performance of the machined workpiece. Compressive RSs inhibit crack propagation and consequently result in a longer fatigue life. Owing to their crucial importance, RSs have gained significant attention over the past few decades and FEM has played a major role in understanding how different machining parameters correlate to RSs. For example, Nasr [6] used Lagrangian FEM to study the effect of sequential cuts on RSs when dry orthogonal cutting AISI 1045 steel, using different feed rates. The predicted results were compared to experimental data. Under the used cutting conditions, surface tensile RSs were generated in the cutting direction and their magnitude dropped with progressive cutting. Furthermore, a larger tool-edge radius was found to induce lower surface tensile RSs. Miguélez et al. [34] used an ALE model to understand the mechanisms behind inducing tensile and compressive RSs. The authors focused on the role of thermal expansion, thermal softening, and the Taylor–Quinney coefficient, controlling the heat generated by plastic flow. They also considered the role of the tool-edge radius. Near-surface tensile RSs were found to increase with higher thermal expansion coefficients, lower thermal softening effects, and when using a larger edge radius. Ozel and Zeren [35] also used ALE to simulate high-speed machining and have found a profound effect for the tool edge on RS and workpiece. Yang and Liu [36] investigated the effect of two different tool–chip friction models on RS when using FEM to simulate the dry orthogonal cutting of AISI 304 stainless steel. In the first model, the friction coefficient was based on the ratio between thrust and cutting force components. In the other model, it was based on the ratio between the average normal and average shear stresses along the tool rake face. When the predicted RS and cutting forces were compared to experimental measurements, it was found that the stress-based friction model is more suitable for simulating the cutting process.

There were several research attempts to estimate the orthogonal cutting parameters inside the metal alloys. For example, several techniques were used to estimate the RSs inside the metal alloys such as X-ray diffraction [6], fiber Bragg grating sensors [37], and the hole-drilling technique. The hole-drilling technique could achieve high accuracy in estimating the RSs against other diffraction techniques, as shown in [18]. Additionally, other research work was carried out to estimate the process parameters in orthogonal cutting of metal alloys such as [38,39,40].

After a comprehensive review of the available literature, it is concluded that the transition from using the J–C damage model to simulate the entire failure process for limiting its role to damage initiation along with using a separate damage evolution criterion has been made without a thorough evaluation. The available literature has mainly reported on the suitability of different models of damage evolution, without comparing their performance to the classical approach, which considers a sudden failure based on the J–C criterion. Furthermore, the effect of damage evolution on FE predictions of RS has not yet been addressed. To the authors’ best knowledge, the first and only currently available attempt made to evaluate the role of damage evolution in FE simulations of metal cutting is the one presented earlier by the current authors in [27]. In [27], dry orthogonal cutting Lagrangian FE simulations were applied on AISI 1045 steel, using a sharp-edged cutting tool. Two damage-modeling approaches were examined; the first used only the pure J–C damage model, i.e., sudden failure, while the second used the J–C damage model for damage-initiation along with a mode I fracture energy for damage evolution. Both approaches were implemented within the sacrificial layer (parting line), one at a time. Moreover, two evolution rates were compared—linear and exponential evolution. Different tests of dry orthogonal cutting were conducted and cutting forces and chip thickness were measured. When the predicted chip thickness and cutting forces were compared to the experimental measurements, it was concluded that damage evolution slightly improved the prediction capabilities. However, such improvements were in the order of experimental error. This insignificant role was attributed to the use of a sharp cutting tool, which is associated with a significantly thin sacrificial layer, along with the very high strain rates encountered in metal cutting. Damage evolution was also found to have an insignificant effect on workpiece temperatures and strains. It is worth noting that the energy-based damage evolution models assumed a constant room-temperature fracture energy value.

The current paper is an extension of the earlier work presented in [27]. It evaluates how damage evolution affects the measurement of RSs, compares the significance of damage evolution when using honed-tools to that of using sharp-tools, and examines the role of temperature in fracture energy-based damage evolution models. A plane strain explicit fully-coupled thermal-mechanical Lagrangian cutting model is built to simulate dry orthogonal cutting using a honed-tool. The workpiece material is of AISI 1045 steel and five different damage models are evaluated. For model validation, dry orthogonal cutting operations are conducted and the measurements of cutting forces, chip thickness, and surface and in-depth RSs are compared to the predicted results. Continuous chips are experimentally generated; however, a failure model is defined within the chip region to ensure that the current models can predict the proper chip type even when a failure model is defined. A damage criterion is used within the parting line and chip region in all five models. The J–C shear failure model is applied as a damage initiation criterion in four cases and to fully model damage (i.e., sudden failure without damage evolution) in one case. Two damage evolution rates are examined. In addition, temperature-dependent and temperature-independent fracture energy-based damage evolution models are compared. In order to evaluate how the tool-edge radius might alter the role of damage evolution, RSs are predicted when using sharp-edged tools using the model presented earlier in [27]. In addition, the sharp-edged cutting forces and chip parameters are re-presented below for completeness and convenience.

## 2. Damage Modelling

### 2.1. Ductile Material Damage

**Figure 1:**
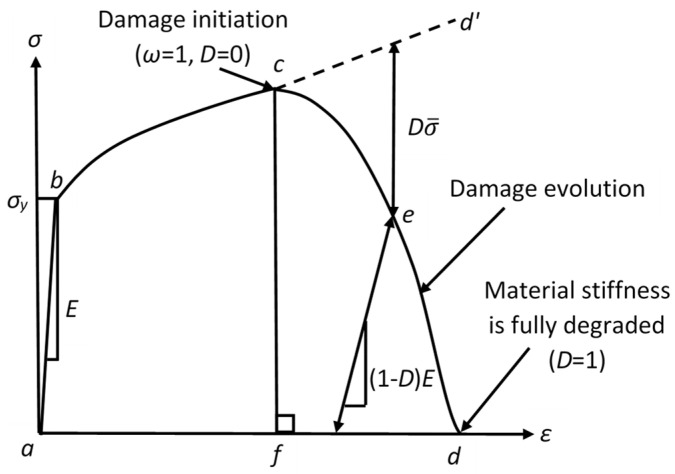
Typical uniaxial engineering stress–strain response of a ductile material.

The ultimate failure of ductile metals occurs over three phases: firstly, the material begins to deform following the material elastic behavior, typically linear (phase one); subsequently, it goes into plastic deformation (phase two) that finally ends with complete fracture (phase three). Figure 1 represents the uniaxial engineering stress–strain curve of a typical ductile material, where straight-line *ab* represents the linear elastic behavior, which eventually reaches the yield strength $\sigma_{y}$, and curve *bc* represents the stable plastic deformation, where *c* corresponds to the instability strain and represents the onset point of damage; i.e., the material starts to lose its load-carrying capacity and the stiffness degradation occurs at point *c*. In Figure 1, curve *abcd’* represents the stress–strain relationship of an intact material that did not include any damage; curve *abcf* represents a material that experienced sudden failure at point *c*; and curve *abcd* represents a material that experienced damage evolution, with a certain rate, after the onset of damage (point *c*). In other words, curve *abcf* is considered a special case of curve *abcd*.

In metal cutting simulations, the J–C constitutive approach is the most widely applied material model for simulating material plasticity (curve *bc*), as it accounts for temperature, strain hardening, and strain rate sensitivity effects [9]. For damage modeling, the J–C damage model [25,41] is widely used; it is either used to fully represent the damage process or accompanied by a damage evolution criterion. When used alone, it corresponds to a case of sudden failure (Figure 1, curve *abdf*), where it does not capture damage evolution. On the other hand, when the J–C damage model is used along with a damage evolution approach, it is only used as a damage initiation criterion; i.e., to represent the onset of damage, while the evolution criterion controls the behavior of the material through curve *cd* (Figure 1). The material loss of its load-carrying capacity is simulated by the stiffness degradation [29]. During damage evolution, the material has a degraded value of Young’s modulus *E’*, which is defined by Equation (1). In Equation (1), *E* is Young’s modulus of the intact material, while *D* is a damage parameter that has a range starting from 0 at no failure to 1 at complete failure. Following the same concept, Equation (2) represents the degraded material flow stress (*σ’*) as a function of the intact material flow stress ($\bar{\sigma }$) [42].

(1) $$E^{\prime }=\left(1-D\right)E$$

(2) $$\sigma^{\prime }=\left(1-D\right)\bar{\sigma }$$

### 2.2. Johnson–Cook Failure Model

As mentioned above, the J–C failure approach is widely used in simulations of metal cutting [27], where it is either utilized alone or accompanied by a damage evolution criterion. The J–C damage criterion depends on the value of the equivalent plastic strain at material failure (${\bar{\varepsilon }}_{ff}^{pl}$), which is defined by Equation (3) [18]. Failure commences when a scalar cumulative damage parameter (ω) in Equation (4) reaches unity [25], at the failure onset (point *c* in Figure 1), ω=1. Applying the J–C failure criterion alone is represented by the vertical straight line (*cf*) in Figure 1, i.e., sudden failure. In Equation (3), *d*_1_–*d*_5_ are material damage parameters; *γ* represents the stress triaxiality ratio, which is the ratio of hydrostatic pressure to Von Mises equivalent stress; $\dot{\bar{\varepsilon }}{\;}^{pl}$ is the equivalent plastic strain rate; ${\dot{\varepsilon }}_{0}$ represents the equivalent reference strain rate; *T* defines the current temperature; *T_r_* is the reference temperature; and *T_m_* is the melting temperature of the material. In Equation (4), $\Delta {\bar{\varepsilon }}^{pl}$ is the equivalent plastic strain increment [25].

(3) $${\bar{\varepsilon }}_{ff}^{pl}=\left(d_{1}+d_{2}e^{d_{3}\gamma }\right)\left(1+d_{4}ln(\frac{\dot{\bar{\varepsilon }}{\;}^{pl}}{{\dot{\varepsilon }}_{0}})\right)\left(1+d_{5}\frac{T-T_{r}}{T_{m}-T_{r}}\right)$$

(4) $$\omega =\Sigma \left(\frac{\Delta {\bar{\varepsilon }}^{pl}}{{\bar{\varepsilon }}_{ff}^{pl}}\right)\;$$

### 2.3. Progressive Damage Criteria

A progressive damage failure criterion is required to simulate the material behavior, specifically the post-failure initiation represented by curve *cd* in Figure 1. The fracture energy-based criteria have been widely used for metal cutting [9,10,32], particularly Hillerborg’s fracture energy criterion [43]. Equation (5) provides the fracture dissipation energy *G_f_* as a function of material yield strength ($\sigma_{y}$); equivalent plastic strain (${\bar{\varepsilon }}_{\;}^{pl}$); and a characteristic length (*L*), which is calculated based on the utilized type of the finite element. The values of *L* for different types of elements could be found in [42]; for first-order elements (used in the current study), *L* is the length of a straight line across the element. In Equation (5), ${\bar{\varepsilon }}_{0}^{pl}$ is the equivalent plastic strain at damage initiation (point *c*), while ${\bar{\varepsilon }}_{f}^{pl}$ is the equivalent plastic strain at complete fracture (point *d*).

(5) $$G_{f}=\int_{{\bar{\varepsilon }}_{0}^{pl}}^{{\bar{\varepsilon }}_{f}^{pl}}L\;\sigma_{y}\;d{\bar{\varepsilon }}^{pl}\;$$

Applying the stress–strain relationship during the damage evolution (curve *cd* in Figure 1), is found not to accurately represent the material post-failure behavior. This is because it is accompanied by a strong mesh dependency, which is attributed to strain localization. Instead, utilizing a stress–displacement response would be able to provide a reliable representation of the material behavior, as it alleviates mesh dependency [11,42]. For that, the equivalent plastic strain (${\bar{\varepsilon }}_{\;}^{pl}$) is replaced by an equivalent plastic displacement (${\bar{u}}^{pl}$) [42] and Equation (5) is modified to Equation (6). The equivalent plastic displacement at failure is represented by ${\bar{u}}_{f}^{pl}$ (point *d* in Figure 1), given by Equation (7), where $\sigma_{y0}$ is the yield strength at the damage initiation state (corresponds to point *c* in Figure 1).

(6) $$G_{f}=\int_{0}^{{\bar{u}}_{f}^{pl}}\sigma_{y}d{\bar{u}}^{pl}$$

(7) $${\bar{u}}_{f}^{pl}=\frac{2G_{f}}{\sigma_{y0}}$$

**Figure 2:**
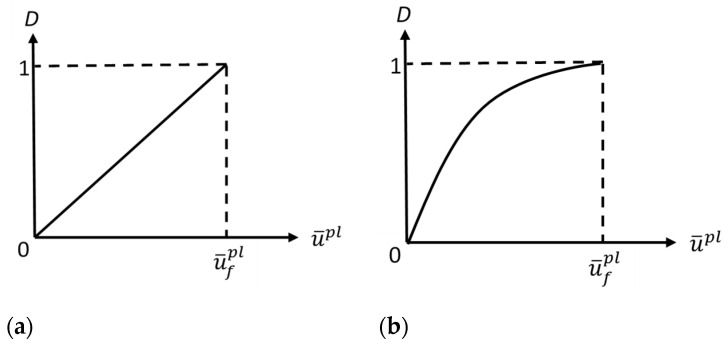
Damage/failure evolution parameter (*D*) in terms of equivalent plastic displacement (${\bar{u}}^{pl}$): (**a**) linear damage evolution and (**b**) exponential damage evolution.

The rate of stiffness degradation/damage evolution (starting from point *c* and ending at point *d* in Figure 1) depends on the change in the damage parameter *D* with the equivalent plastic displacement (${\bar{u}}^{pl}$). Two different damage evolution rates are available in various commercial FE software; that is, linear degradation and exponential degradation [30], as shown in Figure 2. Equation (8) represents linear evolution, where $\dot{\bar{u}}{\;}^{pl}$ is the equivalent plastic displacement rate of change, while Equation (9) represents exponential evolution.

(8) $$\dot{D}=\frac{L\;{\dot{\bar{\varepsilon }}}^{pl}}{{\bar{u}}_{f}^{pl}}=\frac{{\dot{\bar{u}}}^{pl}}{{\bar{u}}_{f}^{pl}}$$

(9) $$D=1-\exp\left(-\int_{0}^{{\bar{u}}^{pl}}\frac{\sigma_{y}{\dot{\bar{u}}}^{pl}}{G_{f}}\right)\;$$

As the fracture dissipation energy *G_f_* is defined by the fracture mode, separation of chip corresponds to an opening mode in the workpiece, and chip serration is defined by the in-plane shear, mode I and mode II of the fracture dissipation energies (*G_fI_* and *G_fII_*, respectively) have been included in the literature to define the damage evolution in the parting line and the chip regions, respectively [26,27,29,32]. Complete failure is achieved when *G_f_* equals the corresponding critical threshold *G_C_*. Equation (10) provides G*_f_* under plane strain conditions, as a function of Poisson’s ratio *v*, modulus of elasticity *E*, and the stress intensity factor *K*. Fracture is developed when *K* reaches the fracture toughness of material *K_C_*. In Equation (10), suffix I corresponds to mode I fracture and suffix II corresponds to mode II fracture. In the present work, the effect of temperature on *G_f_*, and then the cutting simulations, is analyzed using temperature-dependent elasticity modulus. No temperature-dependent data are available for the fracture toughness *K_C_*; accordingly, *K_C_* is assumed to be a constant value (equal to room temperature).

(10) $${\left(G_{f}\right)}_{I,\;II}=\left(\frac{1-v^{2}}{E}\right){\left(K^{2}\right)}_{I,\;II}$$

## 3. Finite Element Analysis

As mentioned earlier, the goal of the current work is to evaluate the role of damage evolution in FE simulations of metal cutting, and how it influences process mechanics and RSs. The current section presents the details of the established FE models, where the role of damage evolution is evaluated during Lagrangian dry orthogonal cutting simulations of steel AISI 1045 (170 HV). Experimental cutting tests (Section 4) are used for model validation.

### 3.1. Model Description

**Figure 3:**
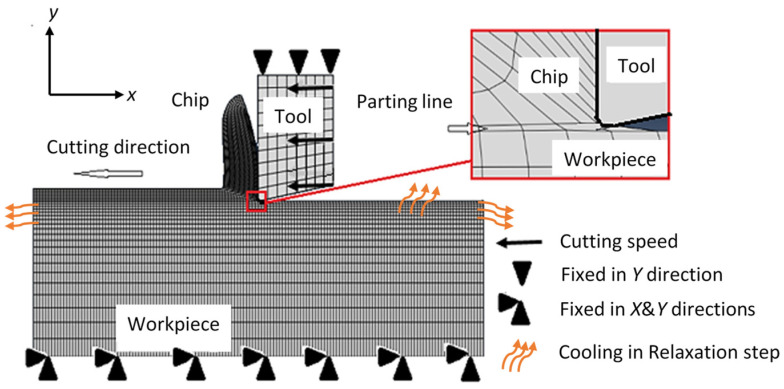
Orthogonal cutting model. Chip formation and damage evolution.

A set of coupled temperature–displacement plane strain Lagrangian models is used to simulate dry orthogonal cutting tests of steel AISI 1045 (170 HV), and subsequently predict RSs. Figure 3 presents the cutting model, which was constructed using Abaqus/Explicit, along with the applied thermal and mechanical boundary conditions. The mesh was generated, refined, and optimized and the model was tested to ensure a minimal effect on the results. A total of five models were developed and used to examine the role of different forms of damage evolution, as detailed below. The experimental cutting conditions, detailed in Section 4, are assigned to the models for validation purposes. Temperature-dependent workpiece mechanical properties are defined, as obtained from [24]. The J–C constitutive equation is used to model the workpiece flow stress. Table 1 lists the J–C plasticity parameters of the used AISI 1045, as found in [27]. For defining the chip formation, a parting line (sacrificial layer) is assumed between the chip region and machined surface, as illustrated in Figure 3. The parting line defined in the honed-tool models is thicker than the sharp-edged models to accommodate the edge radius of the cutting tool. The parting line in the sharp-edged models is 2 µm thick and is not assumed to be 16 µm, the same as the honed-tools, in order to investigate the effect of the coercive increase in the parting line on the predicted results. The workpiece has a 3 mm length and 1 mm height, where the uncut chip thickness is defined as 0.07 mm.

Each model includes two successive simulation steps: firstly, a cutting step is assumed and, secondly, a stress relaxation step is defined, where the second is used to predict RSs. In the relaxations step, the workpiece is left to cool down to ambient temperature and all of the defined boundary conditions are deactivated. A workpiece’s initial temperature of 20 °C is defined prior to cutting, while heat convection to the surroundings is only considered during the relaxation stage, as it is found to have a negligible effect compared with the heat conduction inside the workpiece during cutting [15].

In order to investigate the damage evolution role, five different models were built; Model #1, Model #2, Model #3, Model #4, and Model #5. Model #1 is used as the baseline, which does not consider damage evolution. Further, the J–C failure model (Equations (3) and (4)) is only used, which assumes sudden/sharp failure upon the satisfaction of the criterion (ω=1). However, for the other four models, the J–C failure approach is assumed as a damage initiation criterion and a fracture energy-based approach (Equation (5)) is used to define the damage evolution. Different combinations of the two presently available evolution rates—namely, linear evolution and exponential evolution—are examined and compared. The creation of the chip begins when the parting line is opened, as mode I fracture energy is applied to define damage growth through the parting line. On the other side, mode II fracture energy is applied through the chip region as the chip serration encounters in-plane shear. It is important to mention that continuous chips are experimentally generated under the current cutting conditions (Section 4). However, a damage approach is assumed within the chip to confirm the current model capability to predict the proper chip type even if a damage approach is assumed. Model #2 assumes linear damage evolution in the parting line and exponential damage evolution in the chip region, while Model #3 applies an exponential approach in both areas (parting line and chip). Model #2 and Model #3 include a room temperature fracture energy, i.e., fracture energy is assumed to be independent of temperature. Finally, Model #4 and Model #5 assume similar evolution rates as Model #2 and Model #3, respectively; however, temperature-dependent fracture energy is assumed. As mentioned earlier, only the room temperature *K_C_* values are available. Therefore, effects of temperature on *G_f_* (Equation (9)) are only considered via *E* and *v*. In the present work, *K_IC_* and *K_IIC_* are assigned values of 55.4 MPa.m^1/2^ and 71.5 MPa.m^1/2^, respectively [26]. The implemented J–C damage parameters (*d*_1_–*d*_5_) are reported in Table 2.

### 3.2. Cutting Tool–Workpiece Interaction and Heat Generation

There are two main sources of heat generation in the metal cutting process: (1) friction between the workpiece and tool and (2) workpiece plastic deformation. Surface-to-surface contact pairs are assumed to define the interaction between the workpiece and cutting insert. The Coulomb friction model is assumed and a friction coefficient of 0.2 is assigned along the whole contact length. Even though the actual contact behavior is more complex in nature, the current approach is adopted based on two reasons. First, it has been excessively and successfully utilized in the literature, where reliable results are obtained [2,4,44]. Second, the actual coefficient of friction distribution along the contact length between the tool and chip is very difficult to obtain. Moreover, the value of the currently defined coefficient of friction (0.2) fits well for the range used in the literature when cutting soft materials (similar to the present case) [6,23].

In the current work, all of the generated frictional energy is assigned to be converted into heat energy. The generated heat is then divided between the tool and workpiece depending on their physical properties. Equation (11) defines the frictional heat fraction flowing into the workpiece (*β*), where *β* depends on the effusivity (*Ef*), or heat absorption coefficient, of the workpiece (*Ef_wp_*) and tool (*Ef_t_*). In Equation (11), 𝓀 is the thermal conductivity, *ρ* is the density, and *C_p_* is the specific heat capacity [2,24]. The current analysis considers the heat generation from plastic deformation by assuming 90% of the plastic deformation energy is transformed into heat energy, which agrees well with the work performed in [6,23]. The workpiece’s initial temperature is defined as 20 °C (room temperature).

(11) $$\beta =\frac{Ef_{WP}}{Ef_{WP}+Ef_{T}}\;\mathrm{where}\;Ef=\sqrt{𝓀\rho C_{P}}$$

## 4. Machining Process

**Figure 4:**
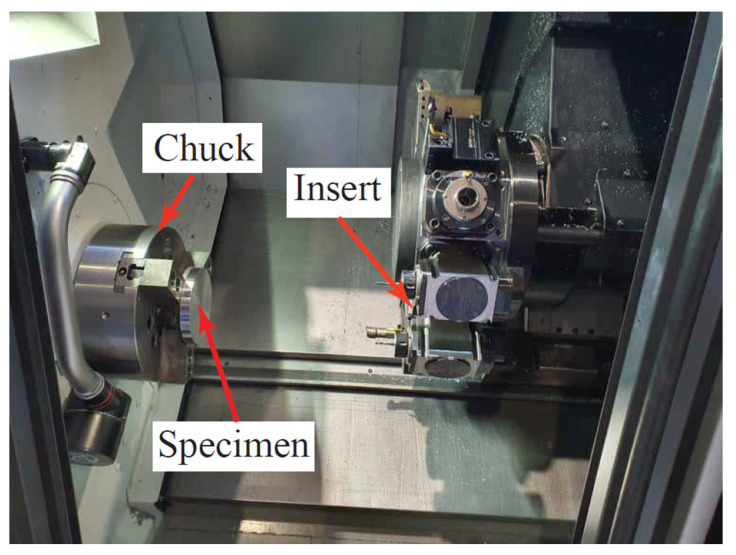
CNC lathe with the AISI 1045 specimen.

This work includes experimental investigations in order to validate the proposed Lagrangian techniques. The experimental tests were carried out using the high-precision CNC lathe (DMG MORI NLX2500/1250), where a set of orthogonal cutting tests are conducted. Figure 4 shows the workpiece mounted in the lathe during the machining operation. The cutting process was applied on steel disks of AISI 1045 (170 HV). The disks had a diameter of 150 mm and a thickness of 30 mm. The material cutting was performed utilizing double-sided rhombic diamond inserts (DNGG 150401-SF). The cutters had a 50 µm edge with a 0° and 11° rake angle and flank angle, respectively. It is worth noting that this was the minimum edge radius for this type of cutter. The cutting operations were performed under dry conditions, using a speed of 100 m/min and a feed rate of 0.07 mm/rev. A small stepover of ¼ of the tool diameter was used in this work owing to the machining of this hard steel specimen as the material was holding small details of RSs and other factors of the cutting process. Generally, the cutting parameters were selected to perform the experimental work for validation and were supported by having a noticeable effect on the investigated outputs, such as RSs, as reported in [6]. The test was conducted several times under the same conditions, while a fresh insert was used for each new cut. It is important to mention that continuous chips were obtained with no signs of phase transformation or built-up edge. The cutting forces were measured using a Kistler dynamometer.

## 5. Measuring Residual Stresses

**Figure 5:**
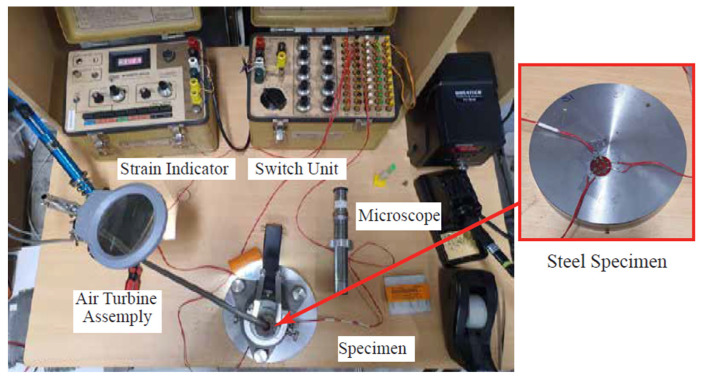
Setup of the hole-drilling method.

The samples were machined and prepared for measuring the induced RSs. The surface and in-depth RSs were measured and analyzed through the direction of cutting. The RSs were measured through the incremental hole-drilling method (IHDM). The IHDM was conducted in three stages, as detailed in [45,46]. A tiny hole was drilled in the area of interest, rosetta strain gauges were used to measure the induced strains, and finally the strains were calibrated numerically and the RSs were measured in the cutting direction. The IHDM apparatus is presented in Figure 5. The drilling speed was set to 20,000 RPM and the feed rate was set to 10 µm/s in the current study. These levels of the drilling speed and feed rate were chosen to achieve the best performance for measuring the stresses based on the optimization work conducted in [47]. The drilling was conducted incrementally and the calibration coefficients were determined through finite element analysis (FEA), as detailed in [13,45].

## 6. Results and Discussion

This section presents the findings of the current analysis, where the damage evolution effects on steady-state RSs in cutting direction (RS11), cutting forces, chip compression ratio (*CCR*), contact length (*l_c_*), and shear angle (*φ*) are presented. Furthermore, the current results are compared to the data obtained when assuming a sharp tool, which were first presented in [27], in order to evaluate the tool-edge radius (*r_n_*) impact on the role of damage evolution and failure propagation in simulations of metal cutting. Three models that include sharp-edged tool are used: Model #6, Model #7, and Model #8, which correspond to Model #1, Model #2, and Model #3, respectively, of the current analysis (honed-tool). It is worth noting that the RS results for the sharp tool are presented here for the first time.

### 6.1. Residual Stresses

**Figure 6:**
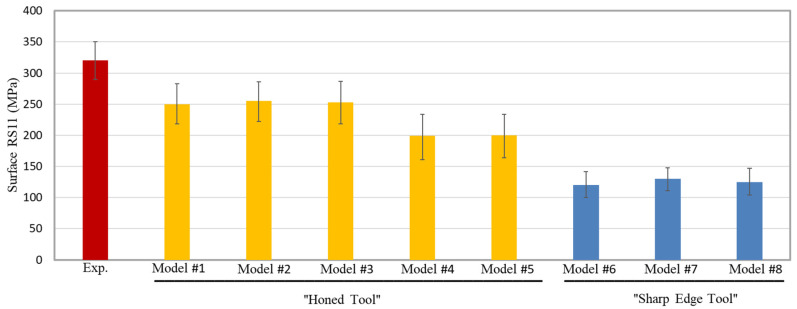
Surface RSs in the cutting direction (RS11)—experimental (exp.) vs. FEA.

Figure 6 compares the predicted surface RS11 for the entire eight models (five using a honed-tool and the three using a sharp-tool) to the experimental IHDM value. In order to exclude the transient effects due to the cutting tool engagement and disengagement with the machined workpiece, the FEA results are extracted from the mid-third of it, which has 50 elements along the length. In Figure 6, the FEA bars represent the average value of the 50 elements and the error bars represent the corresponding standard deviation. As shown, surface tensile RS11 are predicted by all models, which agrees with the experimental finding; however, all models underpredicted the magnitude. As expected, closer predictions are obtained by the honed-edge models, which better represent the experimentally used tool. Sharp-edged models predict lower tensile RSs compared with the honed ones, which agrees with the literature [15]. Lower surface tensile RSs are attributed to lower workpiece temperatures, as shown in Section 6.4, and reflect in lower cutting forces, as shown in Section 6.2. With honed-tools, more material is ploughed into the new machined surface, which results in higher degrees of plastic deformation, which accordingly generate higher temperatures, as demonstrated in Section 6.5. In addition, higher frictional heat is obtained along the honed-edge, which is reflected in the temperature profile along the flank face [15]. The underestimation of surface tensile RSs with the honed-edge models could be attributed to the simple friction model used in the current work, which is incapable of representing the stick-slip model.

The temperature-independent damage evolution models (Model #2 and Model #3) predict almost the same surface RSs, with no significant difference between linear and exponential evolution rates. On the other hand, temperature-dependent damage evolution models (Model #4 and Model #5) predict lower surface RSs owing to the higher fracture energy at a higher temperature. The rise in the fracture energy delays the onset of the failure of the material, which subjects the material behind the tooltip to more tensile strains. Higher tensile plastic strains result in higher compressive or lower tensile RSs [15]. It is worth noting that the variations in the magnitude of surface RS11 are much higher in the honed-tool models as compared with the sharp-edged models. This is attributed to the size of the parting line (sacrificial layer), which is much higher in the case of honed-tools. Thicker parting lines mean more material is removed upon failure, which means higher fluctuations; the same behavior is also noticed in the magnitude of cutting forces.

**Figure 7:**
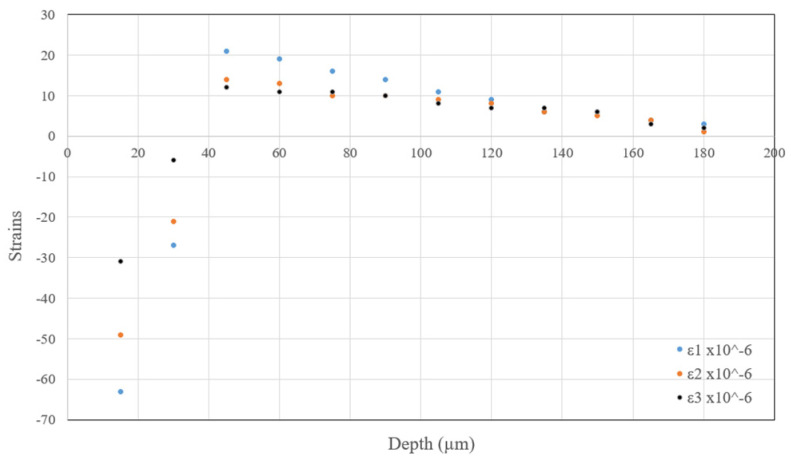
In-depth residual strain distributions in the cutting direction.

**Figure 8:**
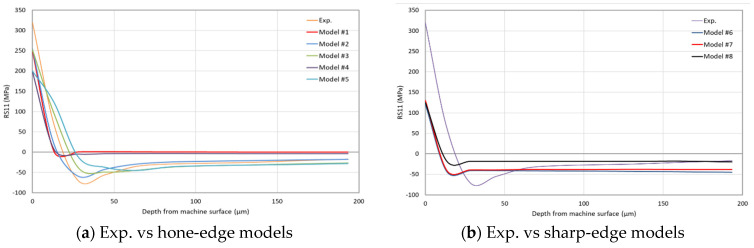

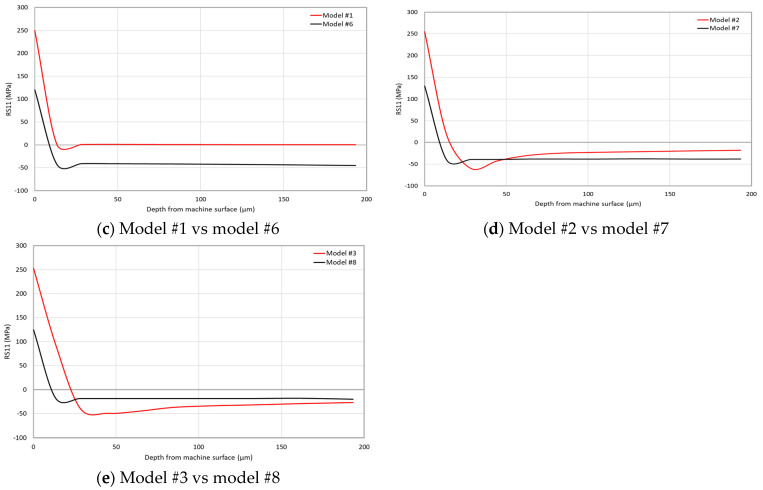
In-depth RSs’ distribution in the cutting direction (*RS11*)—FEA models.

For further examination of the effects caused by the tool-edge radius and the failure models on RSs, Figure 7 shows the average values of strain measurements using the three gauges in the IHDM. The strain results were used to calculate the induced in-depth RSs inside the specimen. Thus, Figure 8a,b show the experimental and predicted in-depth RS11 profiles of honed and sharp edges, respectively. The predicted values in all of the models are lower than the experimental values of the tensile stresses near the surface. The honed-tool models are always closer to the experimental values and are able to predict the location of the peak of compressive stresses, while the sharp-tool models predict the compressive stresses peak earlier than the experimental reference. Additionally, the obtained RS distributions within the depth are close in all of the models. However, the models with temperature-based fracture energy still have lower values than the constant fracture energy models in the upper region near the machined surface. Additionally, the difference between the in-depth distributions of RSs for the linear and exponential softening models is negligible. Figure 8c–e compare between every two similar models from the sharp and honed tools models. The three figures show a difference between the obtained data of the honed-edge and the sharp-tool models, while emphasizing the effect of higher plastic deformation and higher temperature in the models with the curved cutting edge. As mentioned above, the higher plastic deformation caused higher thickness of the tensile RSs in all honed-tool analyses compared with the sharp-edged tools.

### 6.2. Cutting Forces

Table 3 shows the predicted components of the cutting (*F_c_*) and thrust (*F_t_*) forces for the current models, as well as those obtained using a sharp-tool [27], along with the experimentally measured components. The cutting and thrust forces are calculated based on the merchant’s circle theory [48]. The provided values represent the specific force per unit width, in N/mm. In Table 3, the % error represents the difference between the predicted results and the experimental results. For the cutting component (*F_c_*), as expected, the sharp-tool models predict lower values; however, the differences are insignificant. Out of the honed-tool models, the sudden failure model (Model #1) shows the best prediction (1% error), followed by the linear temperature-independent damage evolution model (Model #2), with 4% error; the other three models overestimate *F_c_* by about 15%. The increase in *F_c_* is caused by the delay in achieving the complete damage (D = 1) when damage evolution is considered. For the same reason, higher *F_c_* is predicted by temperature-dependent damage evolution models, as fracture energy increases with temperature.

It is important to note that, as in Table 3, the impact of damage evolution on predicting *F_c_* is more evident when the cutting process uses a honed-tool. This is evident as no significant differences could be observed between the sharp-edge cases, and this can be attributed to the size of the parting line, which needs to be increased with the edge radius. A thicker parting line means more material to be removed (i.e., to achieve complete failure), which requires more energy. The aforementioned effects are supported by the results shown for workpiece temperatures (Section 6.4) and plastic strains (Section 6.5).

The thrust component (*F_t_*) is significantly underestimated by all models, with an absolute minimum error of 66%, even with the honed-tool model. It is important to note that, so far, no explanation has been provided in the literature for the underestimation of *F_t_*, even though it is common when using Lagrangian FE cutting models [15]. It is believed that the underestimation of *F_t_* is mainly due to the inability to properly model material ploughing, particularly when using Lagrangian simulations. It is suggested that using arbitrary Lagrangian–Eulerian (ALE) or coupled Eulerian–Lagrangian (CEL) cutting models could improve the prediction of *F_t_*.

### 6.3. Chip Compression Ratio (CCR), Shear Angle (ф), and Contact Length (l_c_)

Table 4 displays the predicted and experimental results of chip compression ratio (*CCR*), shear angle (*ф*), and contact length (*l_c_*). Firstly, the chip thickness (*t_c_*) is measured, which is then used to calculate the chip compression ratio using Equation (12), where *t* represents the uncut chip thickness. Subsequently, the shear angle is obtained using Equation (13), where *α* refers to the normal rake angle (zero in the present work). Finally, the contact length is calculated using Equation (14). Compared with sudden failure (Model #1), temperature-independent damage evolution models (Model #2 and Model #3) slightly underestimate *CCR* (i.e., thinner chips) and *l_c_* and slightly overestimate *ф*. The opposite takes place with temperature-dependent damage propagation models (Model #4 and Model #5). In general, no significant difference is found between linear and exponential evolution rates. In the case of damage evolution having significant effects, it should have resulted in smaller *ф*, owing to cutting a harder material (delayed damage). The same is expected when comparing temperature-dependent damage evolution models to temperature-independent damage evolution models, because the fracture energy increases with temperature. The current results do not clearly reflect such an expected effect for damage evolution; however, all predicted values are within 11%. Therefore, it is believed that damage evolution has a negligible effect on *CCR*, *ф*, and *l_c_*, as the differences are insignificant, which can be the result of the very limited size of the parting line.

Compared with the experimental results, all models slightly underestimate *CCR* and *l_c_* and slightly overestimate *ф*. Out of the five models, the exponential damage propagation based on the temperature-dependent fracture energy (Model #5) shows the best predictions. It is worth noting that all predictions are within 11% of the experimental results, which is in the order of typical experimental variations. In other words, damage evolution has an insignificant effect on *CCR*, *ф*, and *l_c_* for the examined conditions.

Table 4 also presents the predicted *CCR*, *ф*, and *l_c_* results for sharp-edged models (Model #6, Model #7, and Model #8), as obtained from [27]. In general, the sharp-tool models predicted higher *CCR* (i.e., thicker chips) and *l_c_* and lower *ф*, which agrees with the literature. Compared with sudden failure (Model #6), damage evolution models (Model #7 and Model #8) predict lower *CCR* (i.e., thinner chips) and *l_c_* and higher *ф*. Again, this is opposite to the expected effect; however, again, the differences are insignificant. Therefore, it can be concluded that damage evolution has an insignificant effect on *CCR*, *ф*, and *l_c_*, regardless of the edge radius, for the examined conditions. This can be attributed to the very limited size of the parting line, even in the case of using honed-tools.

(12) $$CCR=\frac{t_{c}}{t}$$

(13) $$\tan\phi =\frac{\cos\alpha }{CCR-\sin\alpha }$$

(14) $$l_{c}=t\;\cdot \;CCR^{1.5}$$

### 6.4. Workpiece Temperature Profiles

**Figure 9:**
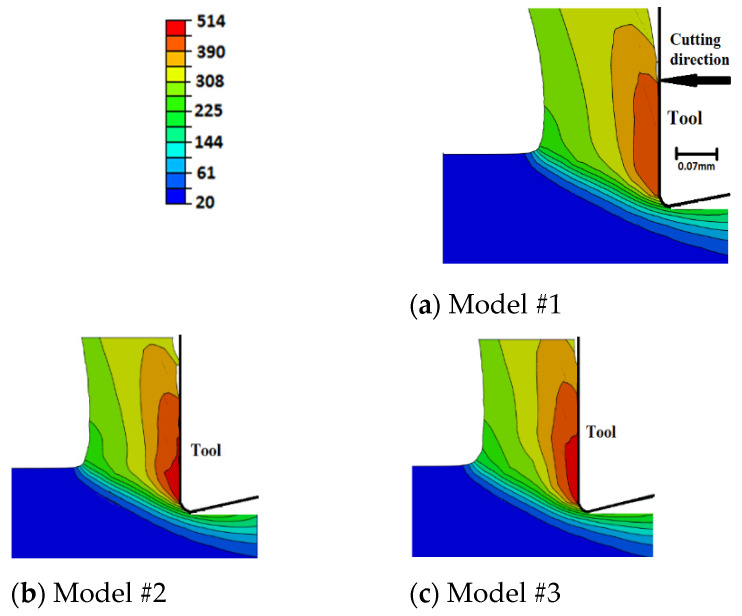

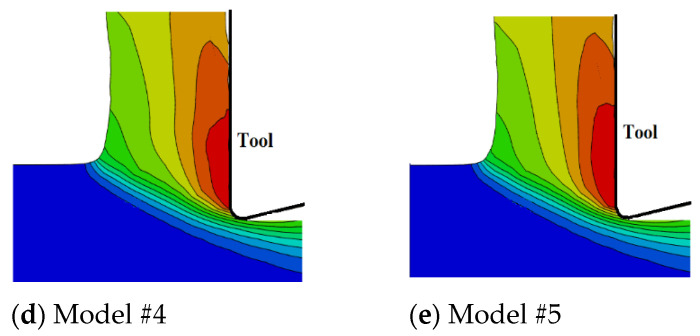
Workpiece temperature (°C) distribution within the chip formation zone—honed tool.

Figure 9 compares the distribution of the workpiece temperature in the chip generation region for the current models. The case of sudden failure (Model #1) predicts lower temperatures compared with the models that include the damage evolution. This is because the instantaneous material losses of its load-carrying capacity make it require less deformation energy and lead to less temperature evolution. On the other hand, temperature-dependent damage evolution models result in slightly higher temperature profiles, with no significant difference between the linear and exponential evolution rates. As the higher temperatures increase the fracture energy, models are included with temperature-dependent damage evolution exhibiting slower damage rates, i.e., higher damage resistance, which is reflected in higher temperature evolution. This is also supported by the slight increase in plastic deformation when using temperature-dependent damage evolution models, as shown in Section 6.5. Overall, Model #5 (exponential damage propagation based on temperature-dependent fracture energy) predicts the highest workpiece temperature, with a maximum difference of about 7% as compared with Model #1 (sudden failure).

**Figure 10:**
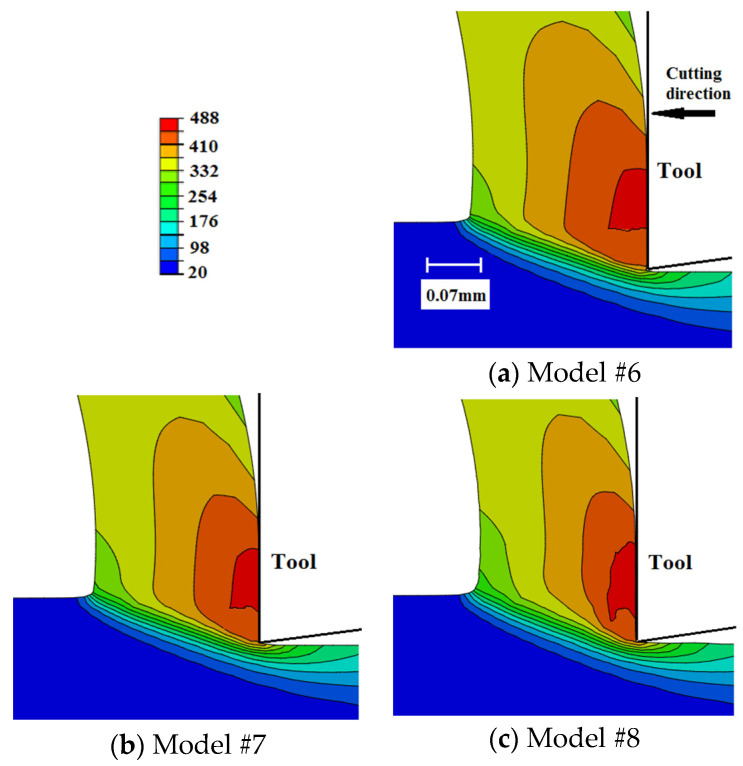
Workpiece temperature (°C) distribution in the chip formation zone—sharp tool [23].

When compared with the predicted temperatures using sharp-edged tools [27], Figure 10, higher workpiece temperatures are generated when using a honed-tool, which agrees with the literature [15]. Furthermore, the damage evolution role is more evident when utilizing honed tools. This is evident as no significant differences could be observed between the sharp-edged cases, i.e., damage evolution has an insignificant effect on workpiece temperatures, which is not the case when a honed tool is used.

### 6.5. Equivalent Plastic Strain

**Figure 11:**
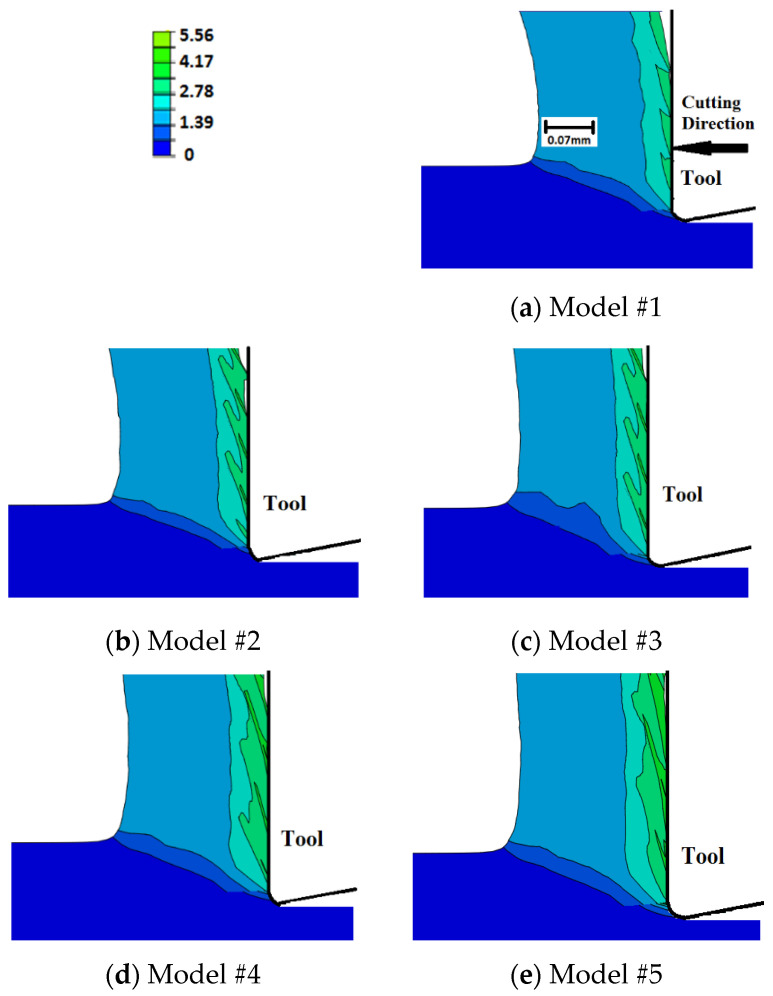
Equivalent plastic strains in the chip generation zone—honed tool.

Figure 11 displays the distribution of equivalent plastic strain (PEEQ) in the region of chip generation for the examined cases. Underneath the tool, damage evolution results in slightly higher plastic strains with higher magnitudes for the exponential evolution rate. Furthermore, the temperature-dependent fracture energy models predict slightly higher plastic strains. Along the rake face, no significant difference is seen in the magnitude of maximum plastic strain; however, a thicker layer of highly deformed material (high PEEQ values) is generated when damage evolution is considered. This is more evident when using temperature-dependent evolution models. All cases experience intermittent distribution of plastic strain along the rake face, with sharper intermittence when damage evolution is not included (Model #1). The intermittent distribution is caused by the element deletion, where a sharp drop in the cutting forces, and accordingly plastic strain and deformation energy, is experienced as an element is deleted. This explains the sharper intermittence when damage evolution is not considered, where elements experience sudden failure/deletion. It is important to note that, as the differences in plastic strains are limited to the very confined zone around the tip of the cutting tool, they have a limited impact on cutting forces. Finally, using temperature-dependent damage evolution models results in slightly higher plastic strains along the rake face, which is attributed to the increase in the fracture energy with higher temperatures, as explained earlier.

**Figure 12:**
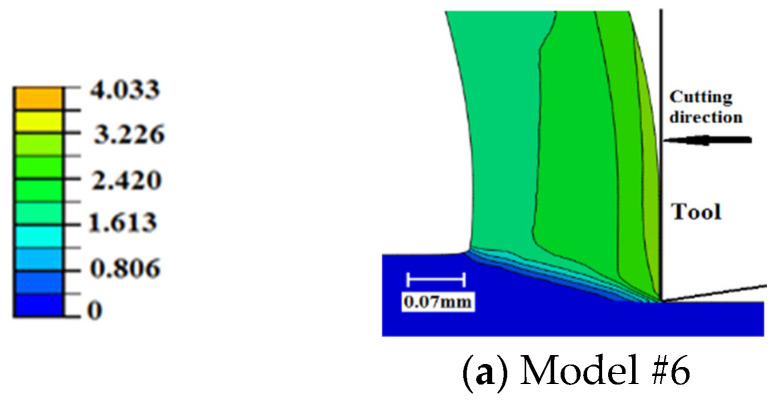

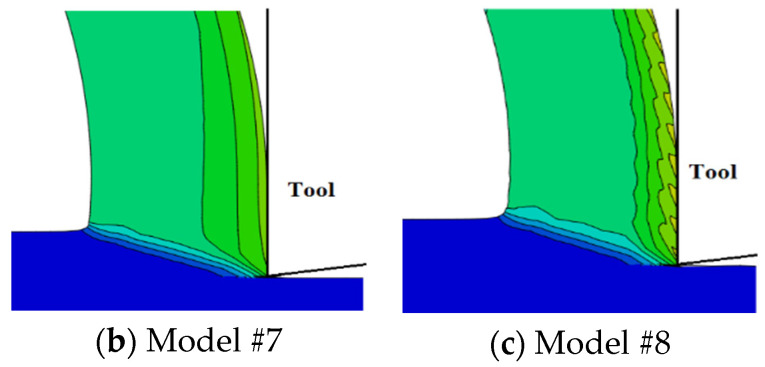
Equivalent plastic strains in the chip generation zone—sharp tool [23].

Comparing the current results to those of sharp-edged tools, Figure 12 [27], it is obvious that using honed tools results in higher magnitudes of plastic deformation, which agrees with the literature [15]. Furthermore, the differences between the three sharp-tool models are less evident than the corresponding models with honed tools, which means that the damage evolution role is less evident when cutting with sharp tools. Moreover, for sharp tools, an intermittent distribution of plastic strain along the rake face is only obvious when exponential damage evolution is used (Model #8). The less significant role of damage evolution when using sharp tools can be attributed to the size of the parting line, which is smaller in the case of sharp tools. A thinner parting line means less material to be removed and, accordingly, the impact of element deletion on plastic deformation becomes less evident.

## 7. Summary and Conclusions

The present analysis examined the role of damage evolution, i.e., progressive damage, in FEA when measuring metal cutting conditions. Dry orthogonal cutting Lagrangian simulations were conducted on steel AISI 1045 (170 HV) using a honed tool. Temperature-dependent and temperature-independent fracture energy models, with linear and exponential damage evolution rates, were examined. At the same time, dry orthogonal cutting tests were performed and the measured results were compared to the predicted ones. The current work focused on surface residual stresses in cutting direction, cutting forces, contact length, chip compression ratio, shear angle, workpiece temperatures, and plastic strain. Moreover, the current results were compared to those obtained when utilizing a sharp cutting tool, which had been presented earlier in [27]. Based on the obtained results, the following conclusions are drawn.

- Surface tensile residual stresses (i.e., 120–255 MPa) were predicted by all models, which agreed with the experimental results. However, all models under-predicted the magnitude. Such an underestimation could be attributed to the simple friction model used in the current work, which was incapable of capturing the stick-slip phenomenon;
- Compared with sudden failure, temperature-independent damage evolution models predicted almost the same magnitude of residual stresses (~255 MPa), with no significant difference between linear and exponential evolution rates. However, temperature-dependent damage evolution models predicted lower surface residual stresses (~200 MPa). This was attributed to the increase in fracture energy with temperature, which delayed the material failure and subjects the material behind the tip of the cutting tool to higher tensile strains. Higher tensile plastic strains resulted in less tensile residual stresses;
- Compared with sharp tools, honed tools provided higher magnitudes of variations in tensile residual stresses. Higher magnitudes were attributed to higher workpiece temperatures. Honed tools resulted in more ploughing, which led to higher degrees of plastic deformation that accordingly generated higher temperatures. Additionally, higher frictional heat was generated when using the honed-edge tools. Higher variations were attributed to the size of the parting line, which was much higher in the case of honed tools. Thicker parting lines mean more material was removed upon failure, which caused higher fluctuations;
- Compared with sudden failure, damage evolution led to higher cutting force components. This was more evident with temperature-dependent damage propagation models and was caused by the delay in achieving complete failure. Furthermore, compared with sharp tools, damage evolution had a more significant effect when using honed tools. This was attributed to the size of the parting line, which was larger for honed tools. A thicker parting line means more material to achieve complete damage before being removed, which requires higher energy and results in higher variations;
- The thrust force component was significantly underestimated by all models with up to 74% deviation from the experimental data. Even though such an underestimation is typical for Lagrangian cutting models, to the best of our knowledge, no explanation has been provided to this phenomenon. It is believed that such an underestimation is mainly due to the inability to properly model the ploughing action underneath the tool tip. Accordingly, it is recommended to use other FEA formulations, such as ALE or CEL, when using honed tools;
- Damage evolution had an insignificant effect on chip compression ratio, shear angle, and contact length regardless of the tool-edge radius, for the examined conditions. This could be attributed to the very limited parting line size, even in the case of using honed tools;
- Compared with sudden failure, damage evolution caused slightly higher workpiece temperatures and plastic strains with a maximum of 514 °C and 5.56, respectively. This was more evident with temperature-dependent damage evolution models. No significant differences were observed between linear and exponential evolution rates. The increase in workpiece temperature and plastic strain was attributed to the increase in deformation energy required to achieve complete failure when damage evolution was considered. Additionally, the increase in the material temperatures caused higher fracture energy, thus the models with temperature-dependent evolution led to a large increase in temperatures and strains. Furthermore, the damage evolution role was more evident when using honed tools. This was, again, attributed to the size of the parting line, as mentioned above;
- Within the chip, no significant differences were noticed in the magnitude of maximum plastic strain. However, a thicker layer of highly deformed material was generated when damage evolution was considered. This was observed with temperature-dependent evolution models. All cases experience intermittent distribution of plastic strain along the rake face, with sharper intermittence when evolution was not considered. The intermittent distribution was attributed to element deletion, which occurred intermittently;
- In general, the current results showed that damage evolution had a limited effect on metal-cutting finite element simulations, which could be attributed to the very limited size of the parting line, even in the case of using honed tools;
- In future work, as an alternative, adaptive meshing techniques, where the parting line does not exist, are planned to be established to simulate the orthogonal cutting processes. Moreover, they will be compared to find the best technique that can predict the cutting process.

## Figures and Tables

**Table 1:** J–C plasticity parameters of AISI 1045 [27].

*A* (MPa)	*B* (MPa)	*n*	*C*	${\dot{\varepsilon }}_{0}\;(s^{-1})$	*m*
553	600	0.234	0.0134	1	1

**Table 2:** Damage parameters of AISI 1045 J–C (unit-less) [27].

*d*_1_	*d*_2_	*d*_3_	*d*_4_	*d*_5_
0.06	3.31	−1.96	0.0018	0.58

**Table 3:** Specific cutting force magnitudes (N/mm).

Force Component	Exp.	FEM							
		Model #1	Model #2	Model #3	Model #4	Model #5	Model #6	Model #7	Model #8
*F_C_*	192	187.4	198.0	218.5	218.3	219.5	179	183	185
% error	-	−1%	4%	15%	15%	16%	−6%	−4%	−3%
*F_t_*	145	38.5	42.1	47.3	46.0	46.4	37.2	37.2	37.8
% error	-	−72%	−70%	−66%	−67%	−67%	−74%	−74%	−73%

**Table 4:** Chip thickness (*tc*), chip compression ratio (*CCR*), shear angle (*ф*), and contact length (*lc*).

Parameters	Exp.	FEM							
		Model #1	Model #2	Model #3	Model #4	Model #5	Model #6	Model #7	Model #8
*t_c_* (mm)	0.220	0.202	0.195	0.194	0.206	0.209	0.248	0.232	0.228
% error	-	−4%	−7%	−8%	−2%	−1%	18%	10%	9%
*CCR*	3.00	2.89	2.80	2.77	2.94	2.98	3.54	3.31	3.26
% error	-	−4%	−7%	−8%	−2%	−1%	18%	10%	9%
*ф* (°)	18.45	19.11	19.70	19.84	18.76	18.92	15.80	16.80	17.00
% error	-	4%	7%	8%	2%	3%	−14%	−9%	−8%
*l_c_* (mm)	0.365	0.343	0.326	0.323	0.353	0.361	0.467	0.421	0.413
% error	-	−6%	−10%	−11%	−3%	−1%	29%	16%	14%

## Data Availability

Not applicable.

## Nomenclature

*A*, *B*, *C*, *n*, *m*	Parameters of the Johnson–Cook plasticity model
*β*	Heat fraction flowing into the workpiece
*CCR*	Chip compression ratio
*C_p_*	Specific heat capacity
*D*	Damage/failure parameter
$\dot{D}$	Rate of change of damage parameter (D)
*d*_1_–*d*_5_	Parameters of the Johnson–Cook failure model
*E*	Young’s modulus for intact material
*E’*	Young’s modulus for damaged/degraded material
*E_wp_, E_t_*	Coefficient of heat absorption of the workpiece and tool, respectively
*F_c_*	Component of cutting force
*F_t_*	Component of thrust force
*G_C_*	Critical fracture dissipation energy
*G_f_*	Fracture energy dissipation
*K*	Stress intensity factor
*K_C_*	Fracture toughness
*L*	Element characteristic length
*lc*	Tool–chip contact length
*r_n_*	Edge radius of cutting tool
*T*	Current material temperature
*T_r_*	Reference temperature
*Tm*	Material melting temperature
*t*	Thickness of uncut chip
${\bar{u}}^{pl}$	Equivalent plastic displacement
${\dot{\bar{u}}}^{pl}$	Rate of change of equivalent plastic displacement
${\bar{\varepsilon }}^{pl}$	Equivalent plastic strain
${\dot{\bar{\varepsilon }}}^{pl}$	Equivalent plastic strain rate
${\dot{\varepsilon }}_{0}$	Reference equivalent strain rate
$\Delta {\bar{\varepsilon }}^{pl}$	Equivalent plastic strain increment
ω	Cumulative damage parameter
*v*	Poisson’s ratio
*ф*	Shear angle
*γ*	Stress triaxiality ratio
*α*	Normal rake angle
$\bar{\sigma }$	Intact material flow stress
$\sigma^{\prime }$	Degraded material flow stress
$\sigma_{y}$	Yield strength
𝓀	Thermal conductivity
*ρ*	Density
Suffix (except if listed above)	
*f*	Failure
0	Material state at the damage onset
I, II	Mode I and mode II of fractures, respectively
